# Global and single-nucleotide resolution detection of 7-methylguanosine in RNA

**DOI:** 10.1080/15476286.2024.2337493

**Published:** 2024-04-02

**Authors:** Silvia D’Ambrosi, Raquel García-Vílchez, Darek Kedra, Patrice Vitali, Nuria Macias-Cámara, Laura Bárcena, Monika Gonzalez-Lopez, Ana M. Aransay, Sabine Dietmann, Antonio Hurtado, Sandra Blanco

**Affiliations:** a Department of Neurosurgery, Cancer Center Amsterdam, Amsterdam UMC, Vrije Universiteit Amsterdam, Amsterdam, The Netherlands; b CIC bioGUNE, Basque Research and Technology Alliance (BRTA), Bizkaia Technology Park, Derio, Spain; c Centro de Investigación del Cáncer and Instituto de Biología Molecular y Celular del Cáncer, Consejo Superior de Investigaciones Científicas (CSIC)-University of Salamanca, Salamanca, Spain; d Instituto de Investigación Biomédica de Salamanca (IBSAL), Hospital Universitario de Salamanca, Salamanca, Spain; e Molecular, Cellular and Developmental Biology unit (MCD), Centre de Biologie Integrative (CBI), University of Toulouse, UPS, CNRS, Toulouse, France; f Centro de Investigación Biomédica en Red de Enfermedades Hepáticas y Digestivas (CIBERehd), Madrid, Spain; g Department of Developmental Biology, Washington University School of Medicine in St. Louis, St. Louis, MO, USA

**Keywords:** 7-methylguanosine, cleavage, deep sequencing, epitranscriptome, RNA modification, transcriptome-wide detection methods

## Abstract

RNA modifications, including *N*-7-methylguanosine (m^7^G), are pivotal in governing RNA stability and gene expression regulation. The accurate detection of internal m^7^G modifications is of paramount significance, given recent associations between altered m^7^G deposition and elevated expression of the methyltransferase METTL1 in various human cancers. The development of robust m^7^G detection techniques has posed a significant challenge in the field of epitranscriptomics. In this study, we introduce two methodologies for the global and accurate identification of m^7^G modifications in human RNA. We introduce borohydride reduction sequencing (Bo-Seq), which provides base resolution mapping of m^7^G modifications. Bo-Seq achieves exceptional performance through the optimization of RNA depurination and scission, involving the strategic use of high concentrations of NaBH_4_, neutral pH and the addition of 7-methylguanosine monophosphate (m^7^GMP) during the reducing reaction. Notably, compared to NaBH_4_-based methods, Bo-Seq enhances the m^7^G detection performance, and simplifies the detection process, eliminating the necessity for intricate chemical steps and reducing the protocol duration. In addition, we present an antibody-based approach, which enables the assessment of m^7^G relative levels across RNA molecules and biological samples, however it should be used with caution due to limitations associated with variations in antibody quality between batches. In summary, our novel approaches address the pressing need for reliable and accessible methods to detect RNA m^7^G methylation in human cells. These advancements hold the potential to catalyse future investigations in the critical field of epitranscriptomics, shedding light on the complex regulatory roles of m^7^G in gene expression and its implications in cancer biology.

## Introduction

In recent years big efforts have been made to elucidate the function of RNA modifications, showing that they play essential roles in regulating RNA metabolism, stability, splicing, localization within the cell and interaction with proteins or other RNAs [1]. Correct deposition of RNA modifications is essential for maintaining translational and transcriptional programmes and for the regulation of several processes including stem cell fate, cell differentiation, migration or response to stress [2–5]. Furthermore, dysregulation of the epitranscriptomic status has been observed in several metabolic, cardiac and neurological disorders as well as in cancer [2,6–9]. Since the development of high-throughput sequencing technologies a new door opened for unravelling the role of these post-transcriptional modifications. High-throughput sequencing methods have not only allowed to map the disposition of specific marks throughout the transcriptome at single-nucleotide resolution, but have also made possible the determination of the reversible and dynamic nature of these modifications under different conditions [10–12]. However, high-throughput sequencing methods have been mainly developed to detect the most abundant mRNA modifications and that is why there is a lack of available methods for the detection of other RNA modifications [10,13].

Over 100 epitranscriptomic marks have been identified in tRNA species, which regulate tRNA processing, folding, stability, and mRNA translation and have important roles in physiology and pathology [4,14–16]. One of the most conserved modifications across species is internal *N*-7-methylguanosine (m^7^G), which is present at the position 46 in the variable loop of some tRNA species. In yeasts, it is catalysed by the complex formed by Trm8 and Trm82 [17], whilst in humans the methylation is deposited by the methyltransferase-like 1 (METTL1) together with the regulatory unit WD Repeat Domain 4 (WDR4) [18]. m^7^G deposition in tRNAs is required for optimal protein translation [18–22], to protect tRNA cleavage [23] and has been described to regulate embryonic stem cells self-renewal and differentiation [18,24]. Furthermore, dysregulation of METTL1 expression levels is associated with different types of tumours such as prostate, colon, lung, gastric, bladder cancer, and glioma [19,22,23,25–30].

In the realm of understanding the intricate web of molecular events governing physiological conditions and their aberrations in various pathologies, the methylation of *N*-7-guanosine (m^7^G) within tRNAs stands as a pivotal enigma. This enigmatic intricacy calls for methodologies that are not only reliable, reproducible, and sensitive but also possess the capacity to decode the precise biological role of m^7^G with unprecedented efficiency. Despite the recent advancements in the development of m^7^G sequencing methods [26,31–34], we are yet to have approaches that allows for quantitative detection of global changes of m^7^G and methods that can precisely map m^7^G sites with high sensitivity.

In this study, we present the optimization of two techniques. The first method harnesses the remarkable specificity of antibodies, affording a global and quantitative analysis of m^7^G in cells, without requiring intricate sample preparation or specialized equipment. What sets our antibody-based approach apart is its remarkable capability to selectively pinpoint m^7^G deposition differences within RNAs derived from biological samples, however it should be used with caution due to variations in antibody quality between batches.

In tandem, we introduce a highly refined sequencing methodology called sodium borohydride (NaBH_4_) reduction sequencing (Bo-Seq). Bo-Seq offers a precise means of mapping m^7^G deposition at the single-nucleotide level, capitalizing on the unique chemical properties of this modified nucleoside. It seamlessly integrates with RNA-seq, setting itself apart by effectively addressing and enhancing the challenges associated with chemical-induced reduction, cleavage, or depurination at internal m^7^G sites. Our method achieves its high reduction, cleavage, and depurination efficiency through a streamlined and optimized protocol that involves high concentrations of NaBH_4_, neutral pH, and the strategic inclusion of 7-methylguanosine as a carrier. This simplifies other similar procedures, reduces protocol duration, and distinguishes Bo-Seq as an efficient alternative to related methods cited in previous studies [26,31–36].

These advancements not only simplify and expedite research efforts but also provide a potent toolkit to unravel the multifaceted roles played by m^7^G in diverse cellular processes and its pertinence in the context of pathologies. The advantages of our methods promise to drive the progress of research in this critical field with unwavering precision and efficiency.

## Results

### Optimization of reducing conditions to efficiently cleave RNA at m^7^G

**Figure 1.**
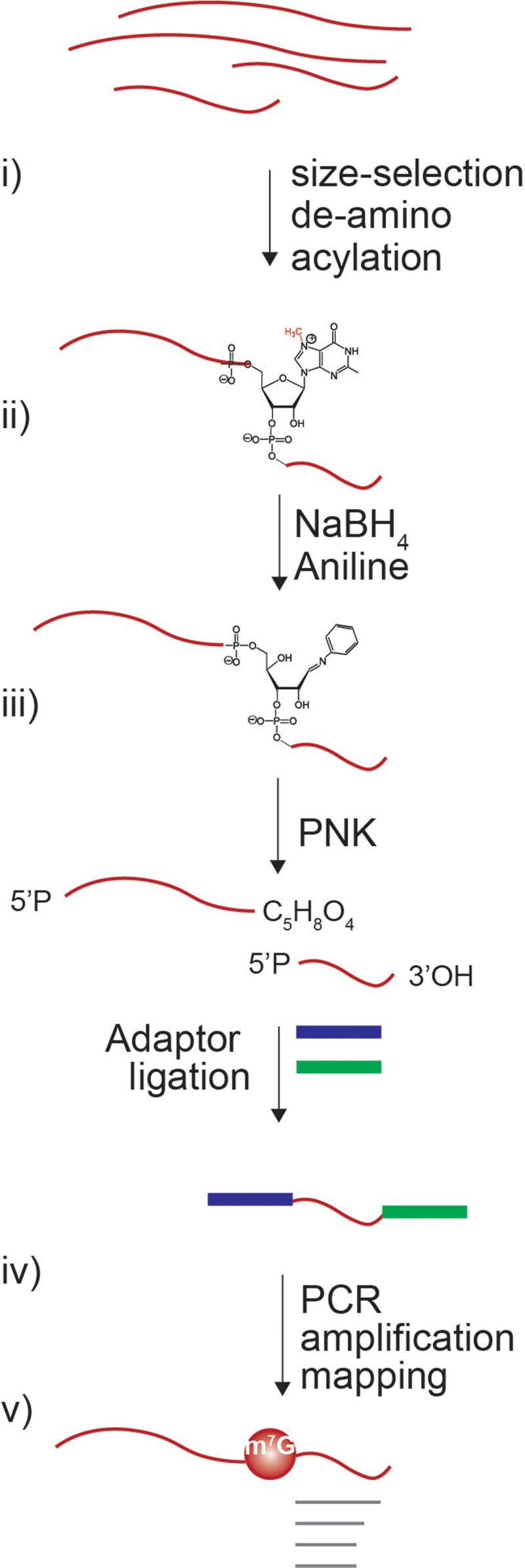
Schematic view of chemistry and Bo-sequencing methodology. Schematic overview of the Bo-Seq method. Representation of the sequential steps: i) size-selection of tRnas and de-aminoacylation; ii) chemical reactions leading to the RNA chain cleavage; iii) 5’ phosphorylation, adaptor ligation (RNA adaptors are indicated in blue and green, RNA in red); iv) library preparation and sequencing; v) bioinformatic analysis (reads are indicated in grey).

m^7^G-modiefied residues are subjected to ring opening via nucleophilic attack by hydroxide anions in reducing conditions, a reaction that ultimately leads to base elimination and the formation of abasic sites [37]. The sequential treatment with aniline leads to the scission of the chain by β-elimination at the N + 1 position at the methylated residue (Figure 1). Chemical reactions using NaBH_4_ and aniline for m^7^G identification in RNA was initially developed to study methylation in synthetic RNAs or RNAs from yeast [38–40]. The method was later applied for DNA and RNA sequencing using Maxam-Gilbert chemistry [41,42]. Additionally, it has been reported that NaBH_4_ can also lead to the formation of abasic sites at position m^3^C and to a lower extent D [32]. However, these modifications are distinguishable from one another as they originate from distinct parental nucleotides. Thus, by exploiting the chemical reactivity of m^7^G to NaBH_4_ mediated reduction, we set to develop a method for globally profiling the m^7^G methylome of RNAs at single-nucleotide resolution. The method proposed in this study would include two main steps: i) the chemical treatment of the RNA to induce the depurination and cleavage of the RNA chain at the m^7^G site; ii) library preparation and sequencing (Figure 1).

To investigate the internal m^7^G profiles within RNA molecules from human, yeast, bacteria, and plants using high-throughput sequencing and at base resolution, various BH_4_-assisted approaches have been proposed [26,31–34]. However, they often fall short in achieving complete cleavage or depurination of internal m^7^G positions, leading to issues with sensitivity and actual proportionality of the overall methylated bases. We attribute this incomplete conversion of internal m^7^G sites during chemical treatment to challenges related to incomplete cleavage or depurination to poor reducing conditions of internal m^7^G sites. In our efforts to enhance the sensitivity, accuracy, and overall performance of our method, we first optimized the most efficient reducing conditions exploring different NaBH_4_ concentrations, pH, as well as the presence of carriers to achieve maximum RNA cleavage at methylated positions. We carefully determined the reduction conditions by conducting a thorough review of the existing literature [38–40]. Our aim was to build upon and enhance the most effective conditions identified in prior studies.

**Figure 2.**
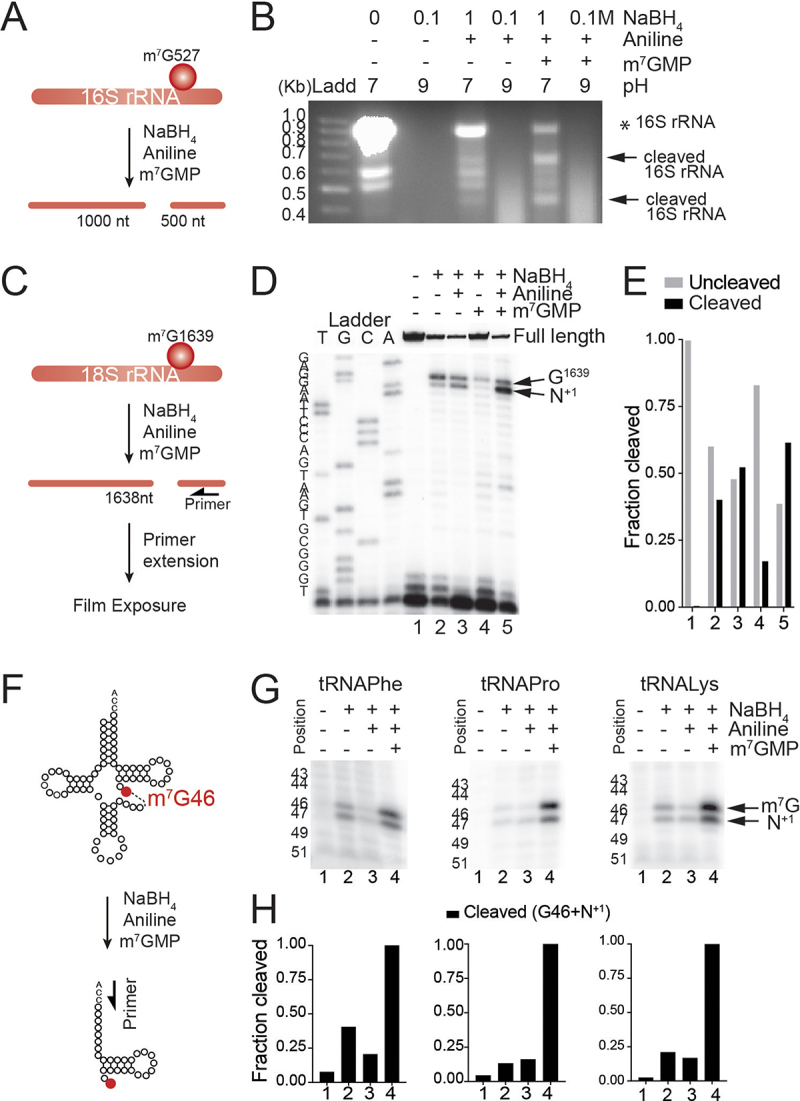
Cleavage at m^7^G is highest when RNAs are treated with 1 M NaBH_4_, aniline and m^7^GMP. (a) schematic representation of prokaryotic 16S rRNA methylated at G527. Treatment with NaBH_4_ and aniline lead to the generation of two RNA fragments. (b) optimization of 16S rRNA cleavage reaction at methylated position m^7^G527 with NaBH_4_ and aniline. The different reaction conditions applied are indicated in the upper panel of the agarose gel. Ladd: ladder. * indicates the full length 16S rRNA and arrows indicate the derived fragments. (c) schematic representation of human 18S rRNA with m^7^G at position G1639. Cleavage of 18S rRNA using NaBH_4_ and aniline lead to the formation of two RNA fragments. Arrow indicates the position of primers in the primer extension reaction. (d) primer extension analysis performed in human 18S rRNA untreated (-) or treated with 1 M NaBH_4_ pH 7.5, aniline and m^7^GMP. (e) each cleaved fraction represents the densitometry of the RT-stop bands relative to the full-length product + each RT-stop (m^7^G + N^+1^ sites) band. The uncleaved fraction represents the densitometry of the full-length product relative to the full-length product + each RT-stop band. (f) schematic representation of tRNA with m^7^G at position G46, and the cleavage that leads to the formation of tRNA fragments. Arrow indicates the position of primers in the primer extension reaction. (g) primer extension analysis performed in human tRnas untreated (-) or treated with NaBH_4_ pH 7.5, aniline and m^7^GMP. The arrow indicates the RT-stops. (h) the fraction cleaved represents the relative densitometry of the sum of each RT-stop bands (m^7^G + N^+1^ sites) relative to the relative densitometry of the sum of each RT-stop bands (m^7^G + N^+1^ sites) in condition 4.

To test and evaluate the efficiency of different reaction setups, we employed *E. coli* 16S ribosomal RNA (rRNA), which is known to carry a unique m^7^G at position G527 [43] (Figure 2(a)). The RNA samples were treated with different concentrations of NaBH_4_ (0.1 M or 1 M), at different pH conditions (pH 7.5 or 9.5), and at the presence of 7-methylguanosine monophosphate (m^7^GMP) as carrier of the reaction according to previous findings [38–40]. The addition of methylated RNA carriers at the reduction stage has been observed to enhance the efficiency of reaction by increasing the yield of cleaved RNAs [40]. According to Zueva *et al.* [40], this effect can be attribute to two main factors: the critical concentration of 7-methylguanosine present in the reaction mixture and its ratio with other nucleotides. In our protocol, we substituted the methylated RNA carriers with m^7^GMP to prevent any possible interference of the RNA carriers on the later library preparation and sequencing analyses, using concentrations recommended by Zueva *et al*. [40]. The β-elimination reaction was performed in all samples using 0.3 M aniline-HCl at pH 4.5.

Scission of 16S rRNA was confirmed by the appearance of the expected two cleaved RNA fragments in the agarose gel (Figure 2(b)). The cleavage at m^7^G position occurred only when the RNA was treated with 1 M NaBH_4_ at pH 7.5 followed by aniline (Figure 2(b), lanes 3 and 5). As anticipated, the presence of m^7^GMP improved the efficiency of reaction, proved by the increase of intensity of the cleaved 16S rRNA fragments (Figure 2(b), lane 5). The reactions performed under alkaline conditions (pH 9.5) led to complete hydrolysis of the rRNA as expected when the reaction is performed in buffers containing Mg^2^+ and Tris [44] (Figure 2(b), lanes 2, 4, 6).

To confirm that the previous optimized setup worked for other RNAs, we applied similar conditions using human RNA. To assess the performance of the reaction, we analysed the formation of a abasic site or the cleavage of 18S rRNA, since it contains a unique m^7^G modification at position G1639 (Figure 2(b)). Because cleavage followed by gel electrophoresis is not a reliable approach, we analysed the cleavage efficiency by primer extension and RT-stop on treated human RNA samples using specific primers designed to validate the cleavage of 18S rRNA at position G1639 (Figure 2(c)). We observed that treatment with NaBH_4_ (with or without the presence of m^7^GMP) led to a creation of an abasic site or a scission which induced a RT stop and at position G1639 (Figure 2(d), lanes 2 and 4) as previously shown [35]. The scission of human 18S rRNA at position G1639 + 1 was specially detected in the treated samples (Figure 2(d,e), lanes 3, 5). The presence of m^7^GMP at the reduction step was confirmed again to increase site-specific fragmentation and depurination of 18S rRNA at G1639 and G1639 + 1 up to over 60% capacity (Figure 2(d,e), lane 5).

To address any differences in sequence context around m^7^G nucleotides between rRNAs and other RNAs, we applied primer extension analysis on treated human tRNA samples using specific primers designed to validate the cleavage of tRNAs at position G46 of known methylated tRNAs; Phenylalanine, Proline and Lysine (Figure 2(f)). We observed the scission of human tRNAs at position G46 and G46 + 1 in the treated samples, and the presence of m^7^GMP was confirmed again to increase site-specific fragmentation of tRNAs (Figure 2(g,h)).

We can conclude that optimizing the reaction by increasing the NaBH_4_ concentration to 1 M, adjusting the pH to 7.5, and adding m^7^GMP significantly enhances the overall m^7^G reduction performance, achieving highest scission/depurination capacity compared to treatment with NaBH_4_. This improvement facilitates cleavage and depurination of RNA at internal m^7^G nucleotides for all tested rRNAs and tRNAs. The scission and depurination sites generated by this chemical treatment can therefore be utilized for the identification of site-specific m^7^G modification sites using high-throughput sequencing technology.

### Single-nucleotide resolution detection of RNA m^7^G

Increasing evidences show that m^7^G disposition on tRNA by METTL1 are involved in important cellular functions and its dysregulation can lead to different diseases, specially cancer [18–22,25–30]. Thus, developing precise and accurate methods to identify m^7^G modification on tRNAs may help to provide insights into the molecular mechanisms and the biological role of this post-transcriptional modification both in physiological and pathological conditions. To enable m^7^G identification at single-nucleotide resolution in tRNAs, we coupled the chemically induced cleavage of m^7^G with RNA size-selection and high-throughput sequencing technology. For this purpose, herein we propose a novel detection technique, named Borohydride-sequencing (Bo-Seq), which incorporates five key steps: i) size-selection of tRNAs and de-aminoacylation; ii) cleavage at N +1 m^7^G nucleotides by NaBH_4_ and aniline treatment resulting in the formation of 3´ fragments with 5’ phosphates; iii) T4 polynucleotide kinase (T4 PNK) phosphorylation to ensure 5’ P and 3’OH ends to enable ligation to RNA adaptors for the construction of sequencing libraries; iv) cDNA library preparation and sequencing; v) bioinformatic analysis (Figure 1).

To benchmark this novel methodology, we investigated the m^7^G methylome of tRNA derived from human cells. As starting material, we size-selected small RNAs using MirVana kit (Thermo Fisher Scientific). Next, we proceed with the de-aminoacylation of mature tRNAs to ensure the presence of aminoacidic-free 3’-ends. De-aminoacylated tRNAs were then split into two groups. Part of the tRNAs were directly used for library preparation as control (‘untreated RNA’). The remaining tRNAs were treated with the previously optimized NaBH_4_, aniline and m^7^GMP reaction to induce the cleavage of the RNA chain at the N +1 m^7^G position by β-elimination. Next, to ensure the presence of 5’P and 3’OH ends for proper library RNA adapters binding to cleaved 3’ fragments and improves the overall adapter ligation efficiency, RNAs were phosphorylated with T4 PNK as in previous protocols for high-throughput tRNA sequencing approaches developed by the group [45]. Next, 3’ ends were ligated to RNA adaptors and reverse transcription was performed using M-MuLV reverse transcriptase, and sequenced each tRNA library at ~10–20 M read depth. Additionally, control libraries prepared with untreated RNAs were used as reference. The protocol can be completed within 4 days.

Next, we focused on validating Bo-seq on finding tRNA m^7^G-methylated positions. Because the analysis of tRNA sequencing is further complicated by the presence of numerous heavily modified bases on tRNA and its intricate secondary structure, we opted to employ a similar tRNA mapping methodology as described by Hoffmann *et al*. [46]. This approach facilitates more accurate tRNA mapping while mitigating errors associated with the structural complexities of modifications (see methodology section). Following this analysis approach, we mapped 535 tRNA genes, included in 48 tRNA isoacceptor families.

**Figure 3.**
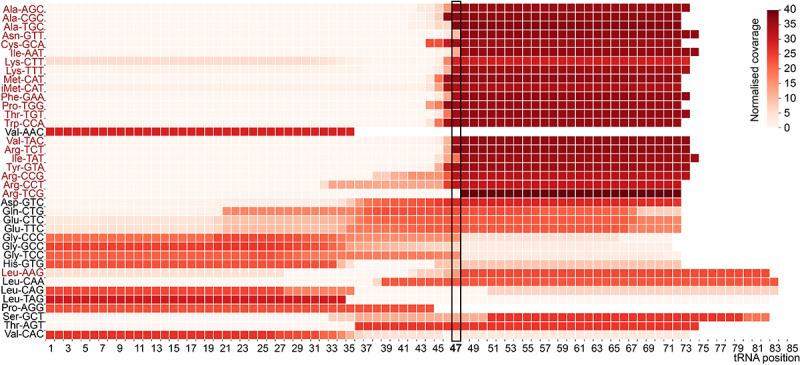
Heatmap showing normalized tRNA fragments read coverage mapped to individual tRNA isoacceptors derived from NaBH_4_-treated RNAs from DU145 cells. Black boxes indicate cleavage site, corresponding to G46 + 1. In red are methylated tRNA isoacceptors.

Mapping the read coverage of tRNA fragments of the treated samples compared with untreated controls indicated specific reverse transcription stops (RT-stop) induced by depurination or cleavage (Figure 3, Supplementary Figure S1). We found that the start site of the RT-stops revealed the location of m^7^G sites from positions N to N + 3, confirming the identification of single consensus methylation sequences within a ~ 4 base region, enabling Bo-seq to achieve high nucleotide resolution detection of the m^7^G modification. In addition, we found, as shown in Figure 3, that the treated samples exhibit a higher coverage of 3’cleaved tRNA fragments than 5’ fragments. This is due to the fact that β-elimination-induced cleavage can lead to the formation of a 5 ‘phosphorylated 3’ fragment at the abasic site and a 4,5-dihydroxy-2-oxovaleraldehyde 3’end attached to the 5’ fragment (Figure 1). Despite using T4 PNK to ensure 5’P and 3’OH ends for adapter ligation, the presence of 4,5-dihydroxy-2-oxo-pentanal at the 3’ end of the 5’fragments interfere with adaptor ligation of these fragments, resulting in the loss of the 5’ fragments in the sequencing libraries [38,47,48], as it happens in other similar methods like TRAC-seq [31]. Because NaBH_4_-aniline treatment could also lead to the formation of abasic sites at 3-methylcytidine (m^3^C), wybutosine (yW) and dihydrouridine (D) positions [49], to avoid off-targets detection, we limited our analysis to the identification of cleavages occurring only at guanosines.

The m^7^G modification sites were identified using a bioinformatic pipeline that calculated a cleavage score at individual sites on tRNAs by comparing the ratio of the number of reads of tRNA fragments that initiate from a specific tRNA internal site in treated samples, to the number of reads occurring across that site in untreated samples (Suplementary Table S1). All bioinformatics analyses employed are freely available at https://github.com/Cancer-Genomics-TH/tRNA_methyl

**Figure 4.**
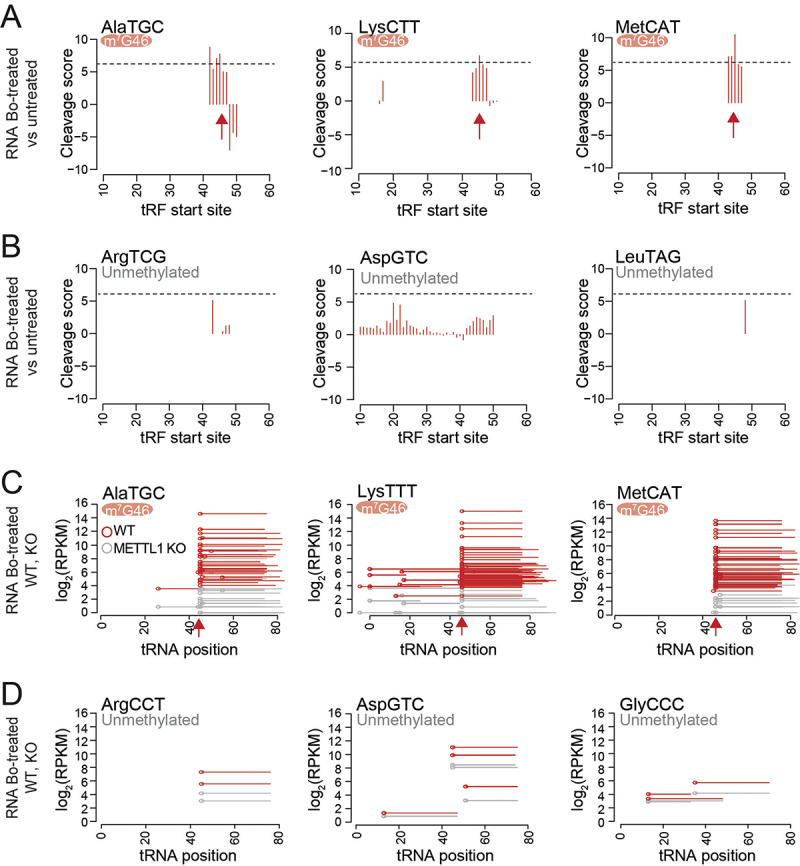
Detection of m^7^G-methylated and unmethylated tRnas using Bo-Seq. A, B) Representative cleavage score profiles of methylated (a) and unmethylated (b) tRnas from DU145 cells. c, d) read coverage of fragmented tRnas after NaBH_4_/Aniline treatment of PC3 METTL1 KO (grey lines) and WT (red lines) RNAs for the indicated m^7^G methylated (c) and non-methylated tRnas (d). Circles represent the tRNA fragment (tRF) start sites. Red arrows indicate cleavage position.

Analysis of the treated RNA sequencing libraries (heatmaps) and cleavage scores higher than 5 confirmed the presence of m^7^G at position G46 in their variable loop in AlaTGC, LysCTT, MetCAT, AsnGTT and ProAGG tRNAs (Figure 4(a), Supplementary figure S2A). Instead, unmethylated tRNA such as ArgTCG, AspGTC, LeuTAG, GluTTC and GluCTC did not show site-specific cleavage after the chemical treatment (Figure 4(b), Supplementary Figure S2B). This cleavage score threshold can be adjusted by the user to enhance confidence or broaden the candidate modification list.

The global profiling of m^7^G deposition in human tRNAs using the Bo-Seq method unveiled the methylation of 22 tRNA isoacceptors at position G46 in the variable loop of tRNAs derived from the prostate cancer cell-line DU145 (Figure 4, Supplementary table S1). Given that METTL1 is responsible of the deposition of m^7^G at position G46 in tRNAs, we compared the m^7^G sites identified by Bo-Seq in DU145 cells with those in the prostate cancer cell-line PC3, in both *METTL1*-expressing (WT) and *METTL1*-lacking (KO) conditions from data published in [23]. Analysis of the cleavage of tRNAs near position 46 of Bo-treated tRNAs from PC3 WT and *METTL1 KO* cells confirmed the presence of m^7^G near position 46 in tRNAs including AlaTGC, AlaCGC, AlaAGC, LysCTT, LysTTT, MetCAT, AsnGTT, ProAGG, PheGAA, among 17 tRNA isoacceptors (Figure 4(c), Supplementary Figure S3A, Supplementary table S2, and [23]. Instead, unmethylated tRNA such as ArgCCT, AspGTC, GlyCCC, ProCGG, ValAAC, HisGTG, did not show site-specific cleavage after the chemical treatment (Figure 4D, Supplementary Figure S3B, Supplementary table S2, and [23]. The Bo-Seq method identified 17 isoacceptors that displayed a loss of methylation upon the removal of *METTL1* in PC3 cells (Supplementary Table S2). Finally, we conducted a comprehensive comparison of the m^7^G sites identified by Bo-Seq with those identified by its most related method TRAC-seq [31]. TRAC-seq method identified 20 isoacceptors, of which 18 were consistent with Bo-seq methylated tRNAs (Supplementary table S2). Thus, this comparative analysis showcased the high performance, sensitivity and specificity of Bo-Seq approach, highlighting its ability to accurately map m^7^G sites in tRNAs.

Therefore, we can conclude that Bo-Seq is a reliable approach for attaining single-base resolution in the detection of m^7^G modifications within human tRNAs. This high-resolution method surpasses other related techniques in reduction performance an demonstrates precision in confirming known methylation sites while providing an opportunity for the identification of novel targets.

### Global detection of RNA m^7^G using antibodies

Despite Bo-Seq allowing for accurate and precise identification of m^7^G deposition at single-nucleotide resolution, it does not allow for a quantitative evaluation of m^7^G deposition levels. Indeed, chemical detection methods are not reliable as quantitative assays due to different factors. To overcome this limitation, we decided to develop a second quantitative detection method to uncover the level of m^7^G transcriptome-wide based on antibody specificity using dot blot analysis.

**Figure 5.**
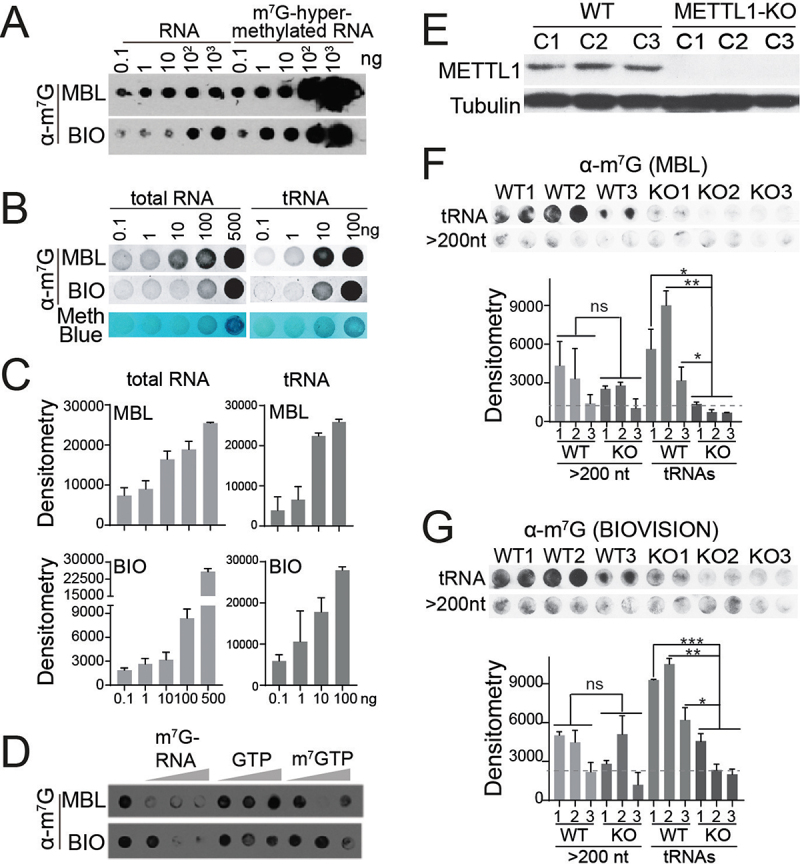
Global detection of m^7^G in tRNA by dot blot. (a) sensitivity of MBL and biovision (BIO) anti-m^7^G antbodies at detecting m^7^G in total RNA. m^7^G-hypermethylated RNA was used as a control. (b) quantitative detection of m^7^G-modified RNAs in increasing levels of total RNA and tRnas from the human cell line PC3 using MBL and biovision (BIO) anti-m^7^G antibodies. One replicate for each condition is shown in the upper panel. Methylene blue staining is used as loading control. (c) bar plots represent densitometry quantification of signal intensity (in arbitrary units) using ImageJ software, normalized to methylene blue. Mean ± SD is represented (*n* = 3). (d) evaluating the specificity of MBL and biovision anti-m^7^G antibodies by competing with m^7^G-hypermethylated RNA (m^7^G-RNA at 20 μg, 200 μg, and 1000 μg), m^7^GTP (at 1 μg, 2 μM, and 4 μM) and GTP (at 1 μM, 2 μM, and 4 μM). E) METTL1 protein expression levels in single-cell derived clones of PC3 control (WT) and PC3 METTL1 KO cell lines. Tubulin was used as loading control. e, f) dot blot assay of tRnas and large RNAs (>200 nt) from PC3 control (WT) and METTL1 KO cell lines after incubation with MBL (e) and biovision (f) anti-m^7^G antibodies. Two technical replicates from three biological replicates are shown for every condition. Densitometry quantification is shown at the bottom. Mean ± SD are represented (*n* = 2). Dotted lines represent average densitometry to all METTL1 KO tRNA samples. Statistics: one-tailed Student’s t-test: *p < 0.05; **p < 0.01; ***p < 0.001.

In order to develop a method that allows relative quantitative detection of global m^7^G deposition in human samples, we first tested the efficiency of two commercially available antibodies from MBL International and Biovision that specifically recognize internal m^7^G marks (Figure 5(a)). Increasing amounts of total human RNA were loaded into nitrocellulose membranes. In addition, m^7^G-hypermethylated human RNAs were generated by dimethylsulfate treatment as indicated in the method section and used as a positive control. As expected, the strongest signal was observed in the hypermethylated samples, validating the ability of the antibodies for recognizing m^7^G-methylated RNAs (Figure 5(a)). Next, to prove the sensitivity of the antibodies to recognize m^7^G-modified RNAs at low levels, we loaded increasing amounts of both total and tRNAs extracted from human PC3 cells. Due to the potential influence of RNA structure, sequence context, and nearby modifications in the vicinity of the m^7^G site on the antibody’s binding capacity to internal m^7^G, we opted for the use of human purified tRNAs instead of *in vitro* synthesized m^7^G-methylated RNA probes. This choice allowed us to optimize exposure time and sample dilution to ensure that the signal falls within the linear detection range of the imaging system when using biological samples. As a result, the increase in signal intensity was proportional to the amount of RNA loaded in all samples, indicating that the use of antibodies to detect modified RNAs from pooled RNA samples allowed for accurate quantification of relative differences between two or more experimental conditions (Figure 5(b,c)). Furthermore, we successfully confirmed the ability of both MBL and Biovision antibodies to detect the m^7^G mark in specific RNA species, particularly tRNAs (Figure 5(b,c)).

An interesting observation arising from our dot blot analysis is the striking similarity in signal detection between tRNA and total RNA samples. This finding strongly suggests that the majority of the signal originates from tRNA rather than from other RNA species. This inference is particularly noteworthy considering the distinct patterns of m^7^G methylation distribution in tRNA and mRNA. While tRNA predominantly contains internal m^7^G modifications, mRNA predominantly contains m^7^G modifications within its cap structure. Nonetheless, despite the potential controversial internal m^7^G in mRNA [32,33,35], our results indicate that the signal detected in total RNA, which encompasses tRNA, rRNA and mRNA, mirrors that of tRNA. This collective evidence underscores the prevalence of internal m^7^G modifications, particularly within tRNA, as the major contributor to the observed signals.

To further prove the sensitivity and specificity of the antibodies, a competition assay was performed. To this aim, the antibodies were previously incubated with increasing concentrations of three different competitors; m^7^G-hypermethylated RNA (m^7^G-RNA), m^7^GTP, and GTP (Figure 5(d)). The signal of detection after the preincubation of both antibodies with the highest concentrations of m^7^G-RNA was dramatically decreased compared to normal signal, but was reduced only modestly after the preincubation of antibodies from Biovision with the highest concentrations of m^7^G-RNA (Figure 5(d)). The signal was also the lowest after the preincubation of antibodies from MBL International with the highest concentrations of m^7^GTP. The signal was unchanged after GTP incubation, indicating that the antibodies specifically recognized the modified nucleoside. In conclusion, while both antibodies seem quite efficient and specific, BML gives a better affinity performance (Figure 5(d)).

To further validate the specificity of the antibodies, we utilized RNA samples from human cells that either expressed (WT) or did not express the METTL1 (*METTL1 KO*). *METTL1 KO* cells were generated using CRISPR/Cas9 technology and the lack of the enzyme was confirmed by western blot (Figure 5(e)). tRNAs and large RNAs (>200 bp), including rRNA and mRNA fractions, from WT and *METTL1 KO* cells, were tested by dot blot with both anti-m^7^G antibodies. A strong signal was detected for tRNAs from WT cells (Figure 5(f,g)). The signal significantly decreased for tRNAs from *METTL1 KO* cells using both antibodies; however, signal decreased was higher for MBL antibody, proving the antibody specificity in biological samples (Figure 5(f,g)). In the large RNA fraction, an extremely subtle signal was detected, which was not significantly changed when comparing WT and *METTL1 KO* RNAs using both antibodies, indicating that most signal was background signal or METTL1-independent (Figure 5(e,f)). These results together suggested that the most specific signal was accomplished with MBL International antibody which resulted from the detection of internal m^7^G.

Thus, we can conclude that the use of MBL International or Biovision antibodies against internal m^7^G in dot blot is a reliable and valuable approach for global detection and relative quantification of m^7^G in different biological samples in a simple and rapid manner.

## Discussion and conclusion

The precise identification of RNA modifications is of paramount importance in understanding their roles in various biological processes and diseases. One such modification, m^7^G, has been implicated in diverse cellular functions, and its dysregulation has been linked to several diseases, including cancer [19,22,23,25–30]. However, the detection of m^7^G modifications at single-nucleotide resolution remains a challenging endeavour. In this study, we have developed and optimized two complementary methods to address this challenge and shed light on the m^7^G methylome of RNA.

### Advantages of Bo-seq

Borohydride-sequencing (Bo-Seq) offers a precise means of characterizing m^7^G tRNA methylomes at single-nucleotide resolution in cell lines, and it holds potential applicability to various tissues and organs. Bo-Seq offers several advantages that distinguish it from related existing techniques [26,31–34]. By carefully selecting the conditions for high NaBH_4_ concentration (1 M), adequate neutral pH (7.5), and the inclusion of m^7^GMP, we have optimized the scission and depurination rates (Figure 2(e,g)), ensuring high performance, sensitivity, and proportionality of m^7^G detection sites, allowing to precisely pinpoint modification sites at the single-nucleotide level. We validate the robustness and reliability of Bo-Seq by successfully detecting all previously reported m^7^G modifications in human tRNA [5,31–33]. Furthermore, another key strengths of Bo-Seq is its simplified workflow. Unlike some existing techniques that involve complex RNA manipulation steps, Bo-Seq streamlines the process, reducing the potential for RNA fragmentation or material loss. This simplicity not only enhances efficiency but also reduces the overall cost and time requirements of the method, making it accessible to a wider range of researchers. Thus, our method arises as a simpler, quicker, cheaper, and reliable technique for precise internal-m^7^G profiling in human tRNAs with high precision and performance.

### Limitations, validation and scope considerations

While Bo-Seq holds great promise in identifying m^7^G sites, it is essential to acknowledge the necessity of validation through independent approaches. Validating newly identified methylation sites is a critical step in ensuring the reliability of our findings, and researchers should exercise caution when interpreting results without this confirmation. It is important to note that our current protocol primarily focuses on the identification of m^7^G sites in tRNAs. While tRNAs play a crucial role in cellular processes, we recognize the need to adapt the method for the detection of m^7^G within mRNA and non-coding RNAs, which may present different challenges and limitations.

### Comparison of Bo-seq with alternative methods

Few other approaches have been developed exploiting the reactivity of m^7^G to NaBH_4_ (Table 1). In comparison with alternative methods, our Bo-Seq approach offers a high performance and a simpler and more streamlined workflow. Many existing techniques involve more elaborate steps, such as RNA enrichment of biotinylated-RNAs as in BoRed-Seq [26], RNA demethylation using recombinant demethylases purified from bacteria which may take up impurities carried in the bacteria preparation, that may induce RNA degradation and loss of input material, or addition of other modifications as in TRAC-seq [31]. These steps can increase complexity and introduce potential pitfalls. Some alternative methods rely on mutational rates for detection, which can introduce variability and affect result accuracy such as m^7^G-MaP-seq, and m^7^G-Seq [33–35]. These approaches bypass the background caused by RNA fragmentation; however, its dependence on the local sequence entail a computational challenge. Furthermore, mutational rates are non-stochiometric, very low and highly variable between replicates, from 5% to 55% mutation rates are incorporated in fully modified positions in RNA from biological samples, potentially leading to a loss of information regarding the extent of methylation at specific sites and, thereby, affecting accurate measurements of the modified sites. Furthermore, other modifications including N2,N2-dimethylguanosine (m^2^_2_G) and N1-methylguanosine (m^1^G) exhibit high misincorporation rates, ranging from 45% to 90% [35]. Since these modified nucleotides share the same parent nucleotide as m^7^G, they can be indistinguishable from m^7^G. In contrast, Bo-Seq directly identifies cleavage and depurination sites with high performance by improving the reducing reaction, avoiding the issue of variable mutational rates and providing consistent and accurate results. RNA modification sites such as D, yW, and m^3^C are also prone to chain scission induced by NaBH_4_/aniline treatment [49]. However, because these modified nucleotides arise from distinct parent nucleotides, they can be easily discriminated during read alignment sing Bo-seq. These characteristics provide the potential for adapting and validating the Bo-Seq method for the detection of other modifications.

**Table 1.** Description of available methods for internal m7G mapping in RNA.

	Method	Type of RNA	Bioinformatics analysis	Advantages	Limitations
m^7^G-MeRIP-seq^33^	Antibody-based approach using anti-m^7^G antibodies	Mammalian tRNA and mRNA	Sequences trimmed with cutadatp2 Alignment done with Tophat2 Reference genome human-hg19 Peak calling with exomePeak and MeTPeak packages Bam-readcount available at https://github.com/genome/bam-readcount RNA-seq data available at GEO GSE112276	Identification of internal m^7^G sites in tRNA and mRNA	Required highly specific antibodies lack of sensitivity to detect m^7^G in low abundant RNA does not uncover m^7^G modification sites at single-nucleotide resolution
m^7^G-MaP-seq^35^	NaBH_4_ treatment leads to formation of abasic sites at m^7^G positions, which are converted into cDNA mutations during reverse transcription process. Increase mutagenesis rate in treated samples compared with controls identifies the m^7^G location	Plant, yeast and human rRNAs and tRNAs	Sequences trimmed with Cutadatp rRNAs from CWR database tRNAs mapped with GtRNAdb Mapping with Bowtie2 Mutation sites identified by mpileup tool Analysis available at https://github.com/jeppevinther/m7g_map_seq Data available at GEO (GSE121927)	It can be applied to different sequencing protocols and allow identification of internal m^7^G position in several RNA types. It bypasses the background caused by RNA fragmentation.	NaBH_4_ treatment increase mutation rate also in ac [4]C, D and m [1]A positions non-stochiometric method high variability between replicates low sensitivity for low abundant RNA and methylated RNA
BoRed-seq^26^	Generation of abasic site using NaBH_4_ at low pH and addition of biotinylated robes to identify methylated RNA	microRNA	Sequenced trimming with Trimmomatic Reference human genome GRCh38/hg38 Mapping with STAR FastQC program available at https://github.com/s-andrews/FastQC Data available at GEO (GSE112180, GSE112181, GSE120454, GSE120455)	Detection of m^7^G in low abundant and small RNA, such as miRNA	Complex steps to add biotin-coupled aldehyde reactive probes
m^7^G-quant-seq^36^	High performance of KBH_4_-induced abasic sites by using high KBH_4_ concentrations, HIV reverse transcriptase and 1 mM dNTPs. Mutations and deletions caused by abasic sites during reverse transcription identify the m^7^G location with quantitative accuracy	Human rRNA and tRNA	Sequences trimmed with cutadatp2 Alignment done with Tophat2 and bowtie2 Reference genome human-hg38 Analysis of mutation rates and deletions Bam-readcount available at https://github.com/genome/bam-readcount RNA-seq data available at GSE209646	Identification of stoichiometric m^7^G modification levels.	complex processing steps and bioinformatic analysis to quantify relative m^7^G levels in tRNA could misidentify m^2^_2_G and m [1]G as m^7^G lack of consistency with other methods identifying only 11 out of the 22 known m^7^G-modified tRNA targets.
m^7^G-seq^33^	Selective biotinylation of NaBH_4_-induced abasic sites. Mutations caused by misincorporation at biotinylated sites during reverse transcription identify the m^7^G location.	Human tRNA and mRNA	Sequences trimmed with cutadatp2 Alignment done with Tophat2 Reference genome human-hg19 Analysis of G→T and G→C mutation rates Bam-readcount available at https://github.com/genome/bam-readcount RNA-seq data available at GEO GSE112276	Identification of low frequency m^7^G modification. It bypasses the background caused by RNA fragmentation	Non-stochiometric method high variability between replicates complex step of biotin conjugation of abasic site using hydrazidation
AlkAlaline-seq^32^	Generation of abasic site under alkaline condition, 5’ and 3’ dephosphorylation followed by aniline-induced cleavage	Bacterial, yeast, and human cytoplasmic and mitochondrial tRNA and rRNA	Raw reads were trimmed using Trimmomatic v32 software Aligment was performed using Bowtie2 (v2.2.4) Code non available RNA-seq data no available	Reliable technique for precise internal-m^7^G profiling in tRNA and rRNA with high specificity	High RNA degradation due to harsh environment limited specificity of the technique due to cross-reactivity with m [3]C and D
TRAC-seq^31^	AlkB treatment of size selected RNA to remove major RNA modifications, followed by NaBH_4_ and aniline treatment	Human and mouse tRNA	Sequences were trimmed using Trim Galore! Aligment was performed using Bowtie tRNAs from GtRNAdb Coverage calculation with Bedtools Analysis available at https://github.com/rnabioinfor/TRAC-Seq RNA-seq data available at GEO (GSE112670)	Specific single-nucleotide resolution profiling of m^7^G sites in tRNAs	long procedure (9 days) demethylation step may lead to loss of input material partially impurities in the recombinant protein may remove modifications or induce RNA degradation non-reliable fragmentation: bias on 3’ tRNA fragment amplification before NaBH_4_ treatment identification of m^7^G sites only tested for small and abundant RNA
Bo-seq	Optimised NaBH_4_-anlinine reaction using adequate concentration of m^7^GMP	Human t RNA	sequence trimming fastp tRNA sequences: predicted by tRNAscan-SE combined with gtRNAdb aligment: LAST isotype position assignments: INFERNAL using tRNA HMM profiles Code available at https://github.com/Cancer-Genomics-TH/tRNA_methyl RNA-seq data available at GEO (GSE203255)	Simple, quick, and reliable technique for precise internal-m^7^G profiling in human small RNAs	identification of m^7^G sites only tested for small and abundant RNA

While our study was under review, Zhang *et al*. published the m^7^G‑quant-seq method in 2022, which also employs BH_4_ reduction and mutation mapping to detect of m^7^G modifications in tRNAs. The m^7^G‑quant-seq method optimizes reduction and depurination rates by using a high KBH_4_ concentration (0.8 M) and HIV reverse transcriptase with 1 mM dNTP concentration. This approach allows for up to 65% conversion of m^7^G to its reduced form in human 18S rRNA and variation rate in tRNAs raging from 54% to 96% depending on the tRNA (e.g. approximately 65% for Phe, 75% for Lys, and 70% for Pro tRNAs). Bo-Seq demonstrates also high performance, achieving maximum reduction capacity in all tested rRNA and tRNA sequences.

One notable advantage of m^7^G-quant-seq, when compared to Bo-seq, lies in its quantitative capabilities. To achieve quantification, m^7^G-quant-seq utilizes calibration curves based on synthetic RNA probes to estimate actual methylation proportions from mutation rates extracted from the sequencing data. However, their validation has been solely reliant on comparisons with data from a separate study that assessed methylation levels in 18S rRNA using mass spectrometry, rather than using their own rRNA data. The absence of other essential controls, such as the examination of methylation rates in tRNAs from biological samples of *METTL1 KO* cells, further raises questions about the quantitative potential of this approach. Additionally, this study does not account for the potentially high mutational rate introduced by m^2^_2_G and m^1^G, which could lead to the misidentification of m^7^G methylation sites. Consequently, this method consistently identifies only 11 out of the 22 known m^7^G-methyalted tRNA. As a result of these limitations and the challenges posed by the calibration curves, the quantitative capacity of m^7^G-quant-seq may not be recommended for accurate assessment of m^7^G modification levels.

In future applications of Bo-Seq, we intend to integrate our protocol with m^7^G-specific antibody immunoprecipitation of RNA fragments prior to NaBH_4_ treatment. This approach has the potential to enhance the detection of sites exhibiting low-frequency m^7^G modifications.

### Quantitative detection with dot blot

In addition to high-resolution detection, we have developed a valuable quantitative antibody-based approach known as dot blot. This method is relatively straightforward and allows for the assessment of relative m^7^G levels in biological samples, offering simplicity, rapid results, and the potential for non-invasive diagnostic applications. Our experiments have demonstrated the sensitivity and specificity of commercially available antibodies against m^7^G, making dot blot a valuable tool for the detection of global m^7^G changes. In addition, we highlight the critical importance of antibody selection in the dot blot approach. While both MBL International and Biovision antibodies have shown efficiency, our experiments indicate that the MBL antibody demonstrates superior performance. This underscores the significance of antibody specificity and sensitivity in achieving accurate and reliable results.

Compare to other methods, this dot blot method for quantitative detection offers a reliable alternative to complex and resource-intensive techniques, such as liquid chromatography with tandem mass spectrometry (LC-MS/MS). LC-MS/MS is the gold standard, but its requirement of high quality and amount of starting material, complexed sample processing steps, advanced and expensive equipment and trained personal limit the technique application [10]. The simplicity, rapidity, and sensitivity of dot blot make it an attractive option for assessing relative m^7^G levels in biological samples. This method stands out for the possibility of directly use low amounts of biological samples (as low as 10 ng), with no need of complex processing into RNA-seq libraries, use of expensive and complex equipment, or application of complex bioinformatic pipelines such as in m^7^G‑quant-seq [36], which could be translated into rapid and easy development of diagnostic kits. m^7^G levels dysregulation is associated with several diseases including cancer [19,22,25–30,36]. Several studies have indicated that increased secretion of m^7^G modified nucleosides can be detected in the urine composition of tumour-bearing rodents and in cancer patients [23,50–54]. Thus, m^7^G deposition detection by dot blot arises as a potential non-invasive diagnostic method to detect relative m^7^G methylation levels in liquid biopsies for rapid and economic evaluation of disease progression.

Regarding this method limitations, antibody-based approaches should be used with caution due to antibody specificity and variations in antibody quality between batches.

In conclusion, our methods represent valuable resources for the study of m^7^G modifications in RNA. Bo-Seq provides high performance at base resolution detection with a simplified workflow, while dot blot offers quantitative assessment. These tools can contribute to a deeper understanding of the role of m^7^G modifications in various biological contexts and diseases, ultimately advancing our knowledge of RNA epitranscriptomics. However, researchers should be mindful of the need for validation and consider the specific scope and limitations of each method when applying them in their studies.

## Materials and methods

### RNA extraction and tRNA size-selection

Total RNA was extracted using Trizol (Honeywell 33,539), followed by DNAse I Turbo (Thermofisher) treatment. Transfer RNAs were size-selected using mirVana small RNA isolation kit (Thermo Fisher Scientific) according to the manufacturer’s instructions. tRNAs were de-aminoacylated in the presence of 1 mM EDTA and 0.1 M Tris-HCl-pH 9.0 for 30 min at 37°C. Concentration was measured using NanoDrop ND-1000. 16s rRNA was kindly provided by Dr Sean R Connell (CIC bioGUNE, Spain).

### Primer extension reactions of human 18S rRNA and tRNA

18S rRNA cleaved after NaBH_4_-aniline treatment was tested by reverse transcription. Briefly, 1 µg of total treated and NaBH_4_-ailine-treated RNA was mixed with 0.2 pmol of a radiolabelled oligonucleotide hybridizing downstream from the expected cleavage site using the reverse primers: 18 sRNA: 5’-ACTTAATCAACGCAAGCTTATG-3’; After heat denaturation (95°C, 1 min), primer extension was carried out with 10 units of Superscript II (Thermo Fisher Scientific) according to the manufacturer’s protocol.

tRNA cleavage was tested by reverse transcription. Briefly, 1.5 µg of total untreated, NaBH_4_ treated or NaBH_4_-aniline-treated RNA was mixed with 0.2 pmol of a radiolabelled oligonucleotide hybridizing at the 3’end of the tRNA, using reverse primers tRNALys: 5’-TGGCGCCCGAACAGGGACTTG-3’; tRNAPhe: 5’-TGGTGCCGAAACCCGGGATCG-3’; tRNAPro: 5’- TGGGGGCTCGTCCGGGATTTG −3’. After heat denaturation (95°C, 1 min), primer extension was carried out with the GoScript™ Reverse Transcriptase (Promega) according to the manufacturer’s protocol. cDNAs were resolved on a 6% polyacrylamide gel. Sequencing, with the ‘rev primer’, of a plasmid containing part of the human 18S rRNA and tRNA sequences were performed with the USB® Sequenase™ version 2.0 DNA Polymerase Kit (Thermo Fisher Scientific) and used as a ladder.

To represent the rRNA cleavage efficiency, we have used the formula: densitometry of each represented band/densitometry of RT-stop band at m^7^G position + densitometry of RT-stop band at m^7^G position + densitometry of full-length product.

Because the detection of full-length products at the top of primer extension northern blots is typically challenging due to the intricate structure and extensive modifications present in tRNAs. These structural complexities often hinder the efficiency of reverse transcription of the full-length product, and only cleaved products are detected. Therefore to represent the tRNA cleavage efficiency, we have used the formula: densitometry of each represented band/densitometry of RT-stop band at m^7^G position + densitometry of RT-stop band at m^7^G position.

### Bo-seq

Libraries were prepared from tRNA from human DU145 cells. m^7^G detection was performed from two replicates of NaBH_4_-aniline treated and non-treated tRNA samples. Total RNA and tRNA were extracted as indicated in the RNA extraction and tRNA size-selection section. 0.1–1 μg of tRNA were treated with 0.2 M Tris-HCl, 0.01 M MgCl_2_, 0.2 M KCl and 10–100 μg of m^7^GMP (Sigma) for 5 min at 85°C according to [40]. Then, freshly prepared NaBH_4_ was added to a final concentration of 0.5 M and samples were incubated for 30 min on ice, and tRNAs were further treated with 0.3 M aniline-HCl at pH 4.5 (Sigma). All small RNAs, included non-treated samples, were incubated with T4 PNK (NEB) to ensure phosphorylated 5’ ends and 3’OH ends. For library preparations, the quantity and quality of the RNAs were evaluated using Qubit microRNA Assay Kit (Thermo Fisher Scientific, Cat.# Q32880) and Agilent RNA 6000 Pico Chips (Agilent Technologies, Cat.# 5067–1513), respectively. Sequencing libraries were prepared following the protocol included with the kit ‘NEXTflex™ Small RNA-Seq Kit v3’ (©Bioo Scientific Corp. Catalog #5132–05). Briefly, RNA of each sample was incubated for 2 minutes at 70°C, then 3’ 4N adenylated adapter and ligase enzyme were added and ligation was conducted by incubation of this mix overnight at 20°C. After excess 3’ adapter removal, 5´-adapter was added alongside with ligase enzyme and the mix was incubated at 20°C for 1 hour. The ligation product was used for the reverse transcription with the M-MuLV Reverse Transcriptase in a thermocycler for 30 min at 42°C and 10 min 90°C. Next, enrichment of the cDNA was performed using PCR cycling: 2 min at 95°C; Cycles of 20 sec at 95°C, 30 sec at 60°C and 15 sec at 72°C; a final elongation of 2 min at 72°C and pause at 4°C. PCR products were resolved on 6% Novex TBE PAGE gels (Cat. # EC6265BOX, Thermo Fisher Scientific), and DNA fragments between 140 bp and 300 bp were cut from the gel. DNA was extracted from polyacrilamide gel using an adapted protocol, in which DNA from gel slices was eluted in nuclease-free water at RT overnight. Afterwards, libraries were visualized on an Agilent 2100 Bioanalyzer using Agilent High Sensitivity DNA kit (Agilent Technologies, Cat. # 5067–4626) and quantified using Qubit dsDNA HS DNA Kit (Thermo Fisher Scientific, Cat. # Q32854). Libraries were sequenced in a HiSeq2500 (Illumina Inc.) until about 15 million 51-bp-reads were obtained.

### m^7^G-methylation analysis

Mapping of NGS reads was done using a constructed genome combining human T2T ver 2.0 sequence (T2T) [55], mature tRNAs from gtRNAdb [56] (hg38 genome) and de novo tRNAScan-SE [57]. We run *tRNAScan-SE* in the appropriate modes using as an input 1. all chromosomes 2. mitochondrial sequence. The predicted tRNA genes were masked in T2T using *bedtools maskfasta* [58]. We attached CCA tails to sequences of tRNA genes from all three sources (gtRNAdb, chromosomal and mitochondrial predictions). Identical (at 100%) tRNA genes/pseudogenes were collapsed using *vsearch* [59]. The obtained unique tRNA sequences were combined with the modified (masked tRNA predictions) T2T genome. The resulting fasta served as a target for mapping NGS reads. The quality checks of FASTQ files was done using *fastp* [60]. This step also removed known primer sequences from the reads. The NGS reads were first clustered using *clumpify* from the *BBMap package* (https://www.osti.gov/biblio/1241166). Read mapping was performed using *LAST aligner* [61]. The MAF files were converted to BAMs and inspected using *samtools* [62] idxstats and viewed in IGV [63]. To filter out pre-tRNas we modified to our needs the method used by Hoffman et al. [46]. In short, FASTQ files were mapped to the unmodified T2T genome extracting read names from 50-bp-long flanking regions (shifted 3bp upstream and 6bp downstream) from the predicted tRNA genes. Next we subtracted these reads present in tRNA genomic flanks from reads mapped to tRNA contigs. After pre-tRNA filtering, hard clipped read sequences were extracted using *samtools*. Only sequences with mapping quality (MAPQ) 1 or higher present in at least 10 separate reads were used. We discarded reads from all tRNA pseudogenes and mitochondrial tRNA contigs. To automate assigning positions corresponding to a given tRNA isoform for fragments mapping to one of the genes of that isoform we applied *cmscan* from *Infernal* [64]. The tRNA alignments were obtained from the HTML page gtRNAdb and converted to Stockholm format. Since the gtRNAdb alignments contained introns we extracted relevant intron-less fasta sequences from the mature gtRNAdb fasta, aligned each isoform with *mlocarna* from the *LOCARNA* package [65]. Final Stockholm file contained therefore both spliced and unspliced alignments. The *cmscan* results (top hits) were then parsed using Python scripts discarding mappings to hmm profiles with introns. The resulting TSV file was used as an input to calculate per base coverages for the heatmaps and cleavage scores.

Whenever practical, several steps i.e. for the construction of the genome, pre-processing and mapping of FASTQ files were combined into Nextflow [66] pipelines.

Cleavage scores were calculated using a custom R script as follows. The cleavage ratio was calculated as the number of RNA fragments with start site i divided by the total number of reads that aligned to that RNA. A cleavage score for site i was calculated as follows: Cleavage score = log2[(cleavage ratio treated RNAs)/(cleavage ratio untreated RNAs)]. Positions with a cleavage score >6.5 are identified as candidate m^7^G sites. Users can modify these thresholds in the programme to redefine the cleavage site criteria. To obtain higher-confidence candidate modification sites, users can increase these thresholds, while reducing them will expand the candidate modification list.

All the code, conda environments, program sources and versions are available from: https://github.com/Cancer-Genomics-TH/tRNA_methyl

### Dot blot

One hundred ng of total, small (<200 nt) and large (>200 nt) RNAs were prepared in 100 µl nuclease-free water and supplemented with RedSafe for visualization. Millipore Milliblot D vacuum kit was employed for loading the samples onto nitrocellulose membranes (HE Healthcare). Next, membranes were left to dry and RNA was crosslinked at 120 mJ/cm^2^ in UV using Stratalinker 2400 (Stratagene). Crosslinked membranes were blocked with 5% BSA in RNAse-free PBS and 0.1% Tween (Sigma) for 1 h at room temperature and then incubated overnight at 4°C in rotation with anti-m^7^G primary antibodies RN017M (MBL) and 6655-30T-BV (Biovision). For competition assays, primary antibodies were incubated one hour at 4°C with increasing amounts of m^7^GTP (NEB), or m^7^G-hypermethylated RNA (Next day, membranes were washed 3 times for 10 min with PBS and 0.1% Tween and then secondary antibodies conjugated with HRP (Cytiva, 1:4000 dilution) were incubated for 1 h at room temperature. Finally, membranes were washed 3 times for 10 min with PBS and 0.1% Tween and signals were developed using ECL (0.1 M Tris-HCl pH 8.5, 0.2 mM coumaric acid, 1.25 mM luminol) and Fujifilm super RX films. Images were digitalized and edited with Image J or Adobe Photoshop. Image J was used to analyse densitometry.

### Methylene blue staining of membranes

Membranes were incubated with 0.04% methylene blue (Sigma) in 0.3 M sodium acetate pH 5.2 (Sigma), until the RNA was stained (approximately 20–30 min). The excess of methylene blue was washed with PBS and 0.1% Tween. Methylene blue staining was used to normalize the signal detected by dot blots.

### RNA m^7^G-hypermethylation

0.3 mg of RNA were resuspended in 90 µl of H_2_O and 10 µl of 10× methylation buffer (500 mM Sodium Phosphate and 10 mM EDTA pH 5.5). Then, 1.7 µl of pure dimethylsulfate (Sigma) was added to the mix and incubated for 5 min at 90°C. The mixture was then transferred on ice, and 25 µl of cold solution containing 1.0 M Tris pH 7.5, 1.5 M sodium acetate, 1.0 M beta-mercaptoethanol (Sigma) and 300 µl of ethanol (Panreac Applichem) were added. The solution was frozen and ethanol-acetate precipitated twice. Methyl-RNA was resuspended in water at 10 mg/ml.

### Cell culture

Human DU145, PC3 were from DSMZ and HEK293FT from Thermofisher. DU145, PC3 and HEK293FT were cultured in DMEM with L-Glutamine and pyruvate (Gibco) supplemented with 10% FBS (Gibco) and 1% Penicillin/streptomycin (Gibco) and maintained in a humidified atmosphere at 37°C and 5% CO_2_. Cell cultures were tested for mycoplasma monthly and maintained mycoplasma-free.

### CRISPR-Cas stable cell lines generation

For knocking-out *METTL1* in PC3 cells, two different sgRNAs (sg117–5’-TATGTCTGCAAACTCCACTTGGG-3’ and sg139: 5’-CAAGTGGAGTTTGCAGACATAGG-3’) were designed with crispor software (http://crispor.tefor.net/) and cloned into the lentiviral vector LentiCRISPR-v2 (Addgene 83,480). A plasmid expressing only Cas9 protein was used to infect control cells. The plasmids were transfected in HEK293FT cells with calcium phosphate together with the appropriate packaging vectors. Viral particles were concentrated using Lenti-X concentrator (Takara) from viral producing cell supernatants 48 and 72 h post-transfection. Viral particles were resuspended in complete medium supplemented with Protamine Sulfate (Sigma), and then added to PC3 cells. Selection of transduced cells was performed with blasticidin (10 µg/mL, Santa Cruz Biotechnology) for 5 days and single-cell clones were generated. Three different clones were selected and validated by western blot, immunofluorescence and genomic DNA sequencing.

### Western Blot

Cells were lysed form 30 min on ice with lysis buffer containing 150 mM NaCl, 40 mM Tris pH 7.6, 1% Triton X-100, 1 mM MgCl_2_ and supplemented with cOmPlete EDTA-Free proteases inhibitor cocktail (Roche) and phosphatases inhibitors 1 mM sodium fluoride, 1 mM sodium orthovanadate and 1 mM β-glycerophosphate (Sigma). After lysis cell debris were cleared by centrifugation for 20 min at maximum speed. Proteins were quantified with Pierce BCA Protein Assay Kit (Thermo Fisher Scientific), separated by SDS-PAGE and transferred to nitrocellulose membranes (HE Healthcare). Blocking was performed for 1 h at RT with 5% skimmed milk in TBS and Tween (0.1%) and primary antibodies anti-METTL1 (Abcam, ab157997) and anti-Tubulin (Abcam, Ab15246) were incubated overnight at 4°C. Membranes were washed three times with TBS-Tween (0.1%) and incubated for 1 hour with secondary antibodies (Cytiva). After three washes with TBS-Tween (0.1%) membranes were developed with ECL (0.1 M Tris-HCl pH 8.5, 0.2 mM coumaric acid, 1.25 mM luminol) using Fujifilm super RX films. Images were digitalized and edited with Image J or Adobe Photoshop.

## Accession numbers

The transcriptomic data generated in this publication have been deposited in NCBI’s Gene Expression Omnibus (GEO) Series accession number GSE203255.

## Code availability

The Bo-seq data analysis source code is available via GitHub at https://github.com/Cancer-Genomics-TH/tRNA_methyl

## Supplementary Material

Supplemental Material
