# Supplementary material for: Global and single-nucleotide resolution detection of 7-methylguanosine in RNA
Source: RNA Biol. 2024 Apr 2;21(1):476–93. doi: 10.1080/15476286.2024.2337493 (PMC10993922; doi:10.1080/15476286.2024.2337493)
Supplement: Supplemental Material [file KRNB_A_2337493_SM7016.zip › Supplementary table 2.pdf]

Supplementary table S2. Comparative table of methylated tRNAs

tNA type	tRNA isoacceptor	Methyalted by Bo-seq in DU145 cells (this paper)	Methyalted by Bo-seq by METTL1 in PC3 cells (doi: 10.1038/s41388-023-02825-0	Methylated by TRAC-seq
Ala	AGC	Yes	Yes	Yes
Ala	CGC	Yes	Yes	Yes
Ala	TGC	Yes	Yes	Yes
Arg	ACG			
Arg	CCG	Yes		
Arg	CCT	Yes		
Arg	TCG	Yes		
Arg	TCT	Yes	Yes	Yes
Asn	GTT	Yes	Yes	Yes
Asp	GTC			
Cys	GCA	Yes	Yes	Yes
Gln	CTG			
Gln	TTG			
Glu	CTC			
Glu	TTC			
Gly	GCC			
Gly	CCC			
Gly	TCC			
His	GTC	Yes		
Ile	AAT		Yes	Yes
Ile	GAT			
Ile	TAT	Yes	Yes	
Leu	AGC			
Leu	AAG	Yes		
Leu	CAA			
Leu	CAG			
Leu	TAG			
Leu	TAA			

Lys	CTT	Yes	Yes	Yes
Lys	TTT	Yes	Yes	Yes
(i)Met	CAT	Yes	Yes	Yes
Phe	GAA	Yes	Yes	Yes
Pro	AGG	Yes	Yes	Yes
Pro	CGG			Yes
Pro	TGG	Yes	Yes	Yes
Sec	TCA			
Ser	ACT			
Ser	AGA			
Ser	CGA			
Ser	GCT			
Ser	TGA			
Thr	AGT			
Thr	CGT			
Thr	TGT	Yes	Yes	Yes
Trp	CCA	Yes	Yes	Yes
Tyr	GAT	Yes	Yes	Yes
Val	AAC			Yes
Val	CAC	Yes		Yes
Val	TAC	Yes	Yes	Yes
