# Supplementary figures and images for: Global and single-nucleotide resolution detection of 7-methylguanosine in RNA
Source: RNA Biol. 2024 Apr 2;21(1):476–93. doi: 10.1080/15476286.2024.2337493 (PMC10993922; doi:10.1080/15476286.2024.2337493)

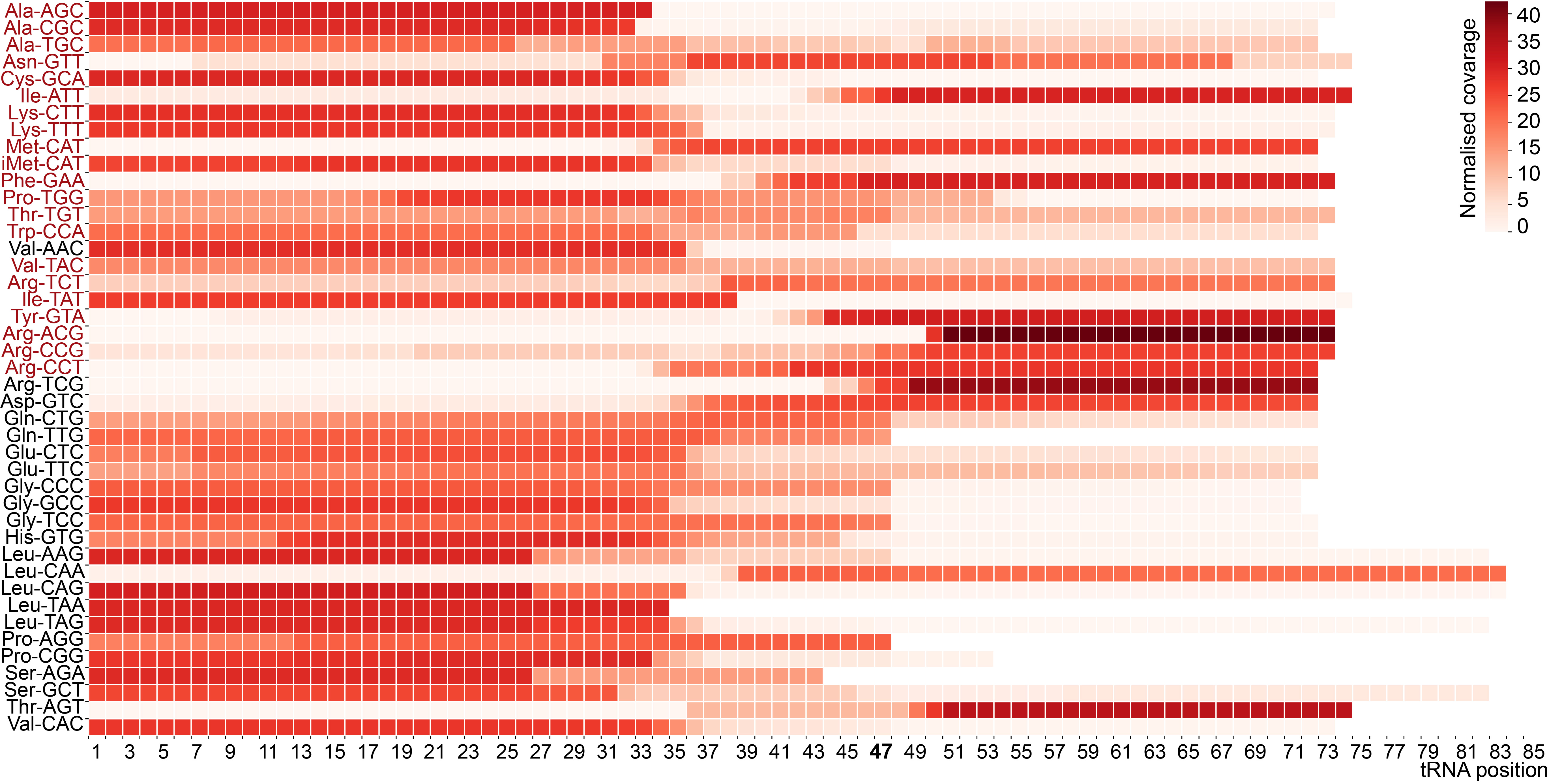

Supplement: Supplemental Material [file KRNB_A_2337493_SM7016.zip › Supplementary Figure 1.jpg]

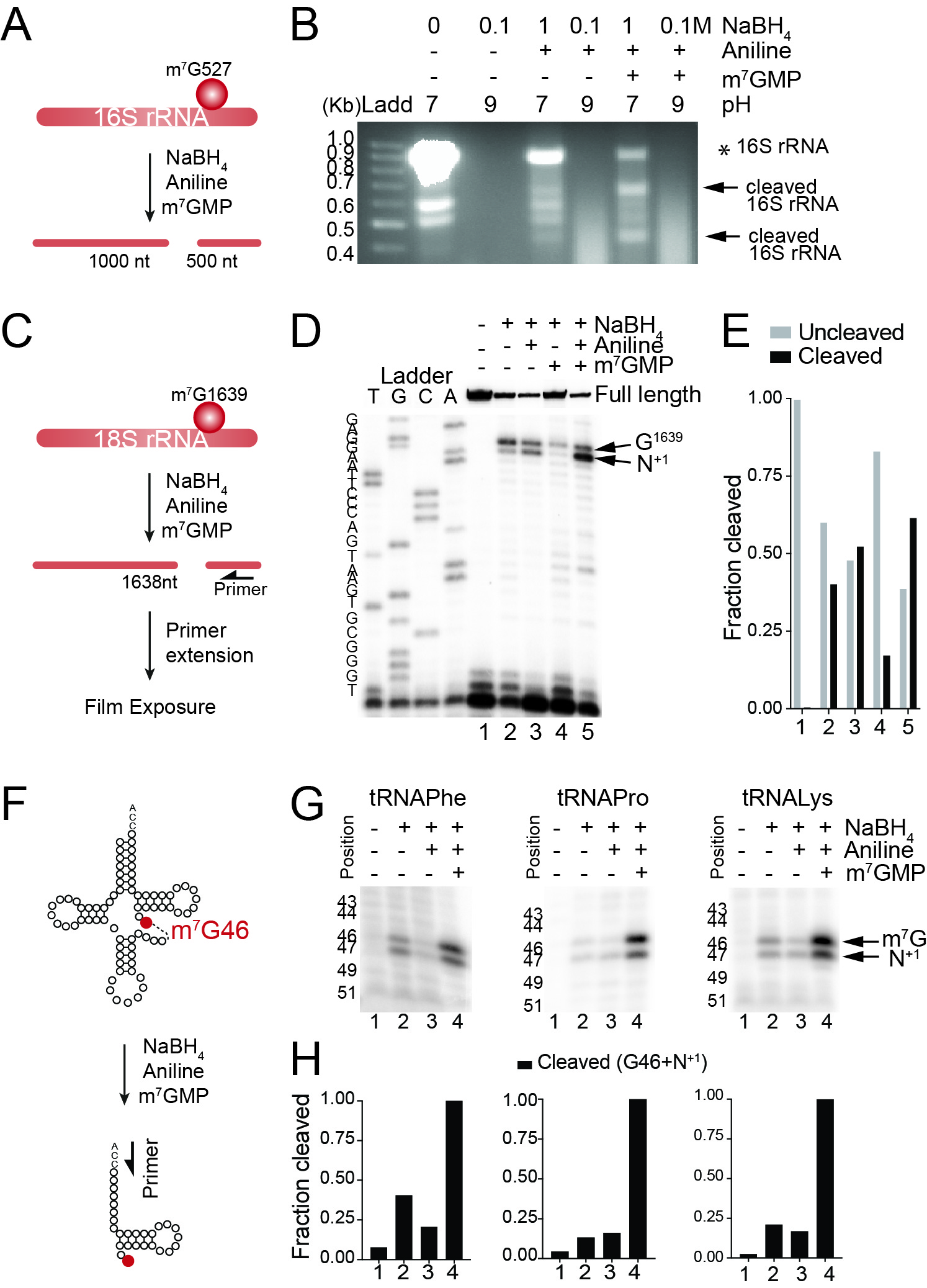

Supplement: Supplemental Material [file KRNB_A_2337493_SM7016.zip › Supplementary Figure 2.jpg]

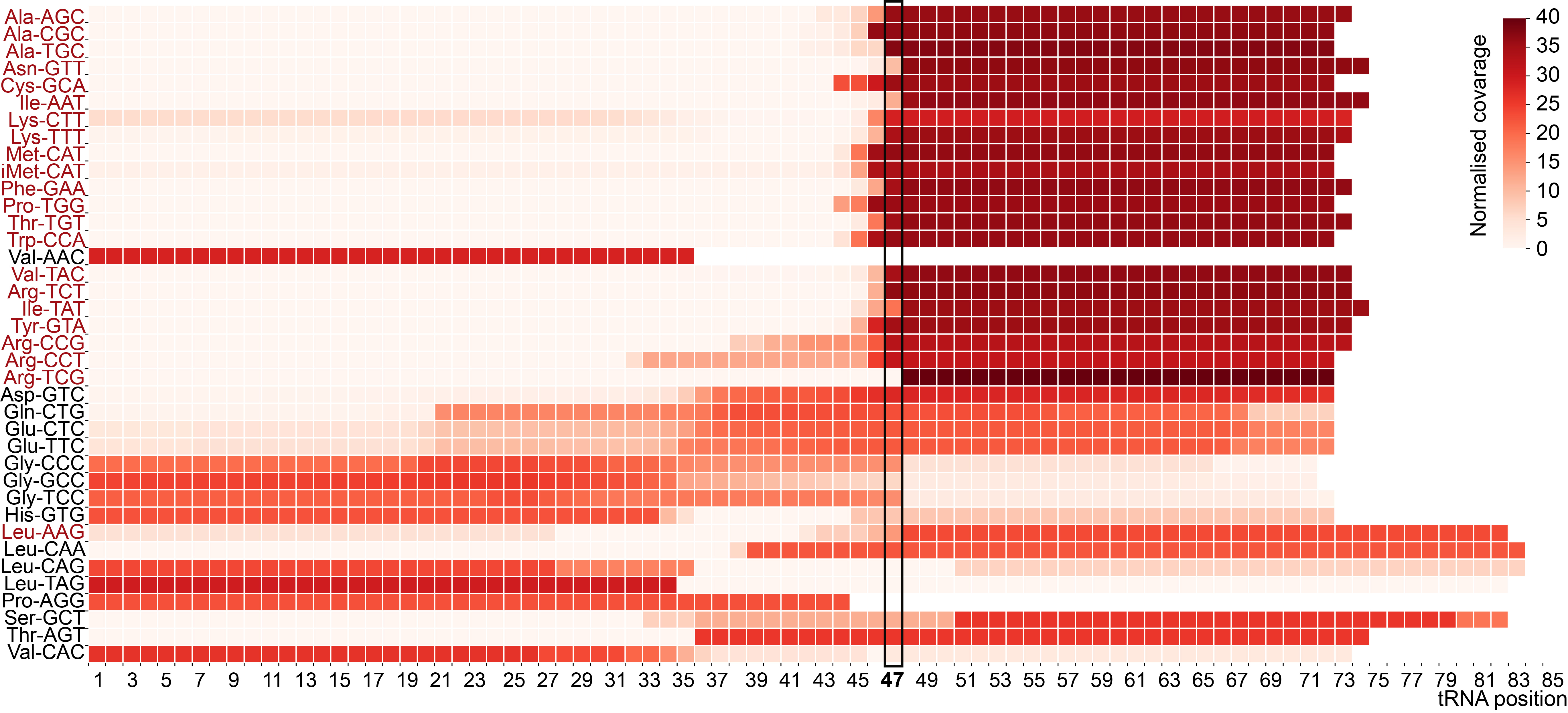

Supplement: Supplemental Material [file KRNB_A_2337493_SM7016.zip › Supplementary Figure 3.jpg]
